# Association of the monocyte-to-albumin ratio with cardiovascular disease and with all-cause and cardiovascular mortality in the general population

**DOI:** 10.3389/fcvm.2025.1645793

**Published:** 2025-09-22

**Authors:** Yingju Jin, Xiaoyue Wang, Yinlian Chen, Xiaomei Li, Xueqing Wu, Yunxue Tian, Juan Li

**Affiliations:** ^1^ School of Nursing, Guizhou University of Traditional Chinese Medicine, Guiyang, China; ^2^ Public Health School, Zunyi Medical University, Zunyi, China; ^3^ School of Nursing, Zunyi Medical University, Zunyi, China; ^4^ School of Nursing, Guizhou Medical University, Guiyang, China; ^5^ Department of Nursing, Guizhou Provincial People’s Hospital, Guiyang, China

**Keywords:** monocyte-to-albumin ratio, cardiovascular disease, all-cause mortality, cardiovascular mortality, NHANES

## Abstract

**Objective:**

This investigation examined the links of monocyte-to-albumin ratio (MAR) with cardiovascular disease (CVD), alongside all-cause and CVD mortality in the general population, employing records from the National Health and Nutrition Examination Survey (NHANES) conducted between 2001 and 2018, connected to the National Death Index (NDI).

**Methods:**

Participants were split into three cohorts based on MAR. The link between MAR and CVD was examined by multivariable logistic regression analysis. Curve-fitting techniques investigated potential nonlinear associations. Receiver operating characteristic (ROC) curves determined the predictive capability of MAR regarding CVD. The links of MAR with all-cause and CVD mortality were investigated utilizing Cox regression modeling. Restricted cubic splines (RCS) curves illustrated the dose-dependent relationships. The weighted Kaplan–Meier method assessed survival distinctions among MAR tertile categories. Sensitivity and stratified analyses were performed to assess the robustness and stability of the results. Further, NRI analysis was conducted to quantify the predictive performance of MAR.

**Results:**

Multivariable logistic regression identified a notable positive link between MAR and CVD (*p* < 0.05), with a nonlinear trend confirmed by smoothing curves (nonlinear *p* = 0.019). Subgroup analyses indicated the consistency of this association (interaction *p* > 0.05). Through ROC evaluation, MAR exhibited robust predictive capability for CVD. Increased MAR measurements correlated with heightened risk of all-cause mortality [hazard ratio (HR): 1.25, 95% CI: 1.08–1.44] and CVD mortality (HR: 1.46, 95% CI: 1.10–1.93). RCS evaluation determined a nonlinear connection between MAR and all-cause mortality (nonlinear *p* < 0.05). The results maintained stability throughout sensitivity and stratified assessments.

**Conclusions:**

MAR demonstrated a positive correlation with CVD, and higher MAR levels were notably connected to an occurrence rate of both all-cause and CVD mortality. Further exploration of the feasibility and predictive capabilities of MAR as an emerging inflammatory marker is warranted.

## 1 Introduction

With the accelerating global aging trend, the incidence and mortality of cardiovascular disease (CVD) continue to rise, posing a significant public health challenge worldwide (1). Despite advancements in prevention and treatment, CVD remains the leading cause of death globally (2). Timely identification of additional risk factors is therefore essential to prevent, delay, or reduce the onset, progression, and mortality associated with CVD. Inflammation has emerged as a key determinants in CVD, serving a pivotal function in its development and prognosis (3). Current research demonstrates the forecasting and outcome-indicating significance of integrated inflammatory indicators, encompassing the monocyte-to-albumin ratio (MAR), neutrophil-to-lymphocyte ratio (NLR), systemic immune-inflammation index (SII), and monocyte-to-lymphocyte ratio (MLR) for CVD (4–7).

MAR, a novel inflammatory-nutritional marker, has attracted increasing attention (8). However, current research on MAR has primarily focused on chronic disease settings. For example, Zhao et al. (9) reported that MAR levels were significantly elevated in patients with non-small cell lung cancer (NSCLC) compared with healthy controls, suggesting that MAR may serve as a novel auxiliary biomarker for NSCLC detection. Similarly, Cao et al. (10) observed that MAR levels were positively associated with the prevalence of chronic obstructive pulmonary disease (COPD). In another NHANES-based study, elevated MAR was found to predict an increased risk of all-cause mortality among individuals with Parkinson's disease (11). These studies highlight the potential prognostic value of MAR in chronic disease conditions. Nevertheless, its role in acute pathological processes remains unclear, particularly in the context of cardiovascular events such as stroke and acute myocardial infarction, for which current evidence is still lacking.

MAR reflects the balance between monocyte count and albumin levels. Monocyte-mediated inflammatory responses are central to the pathogenesis of atherosclerosis and its associated CVDs, demonstrating a heightened sensitivity to even minor alterations in cardiovascular function (12). Serum albumin levels contribute to CVD primarily through their anti-inflammatory, antioxidant, and antithrombotic properties (13–16). Additionally, albumin serves essential functions in molecular transportation and osmotic balance, potentially safeguarding against various CVDs (17). MAR, as a composite blood-based indicator integrating monocyte count and serum albumin levels, is minimally affected by physiological fluctuations, thereby providing comprehensive information and exhibiting considerable predictive potential (18). However, current evidence regarding MAR in cardiovascular-related acute diseases is limited, and its predictive value for CVD and mortality outcomes in the general population remains unclear. Thus, the investigation seeks to assess the correlation between MAR and CVD, alongside total and cardiovascular mortality rates within the general population, examining its applicability as an innovative biomarker for early detection and outcome assessment of CVD.

## 2 Materials and methods

### 2.1 Study design and population

**Figure 1:**
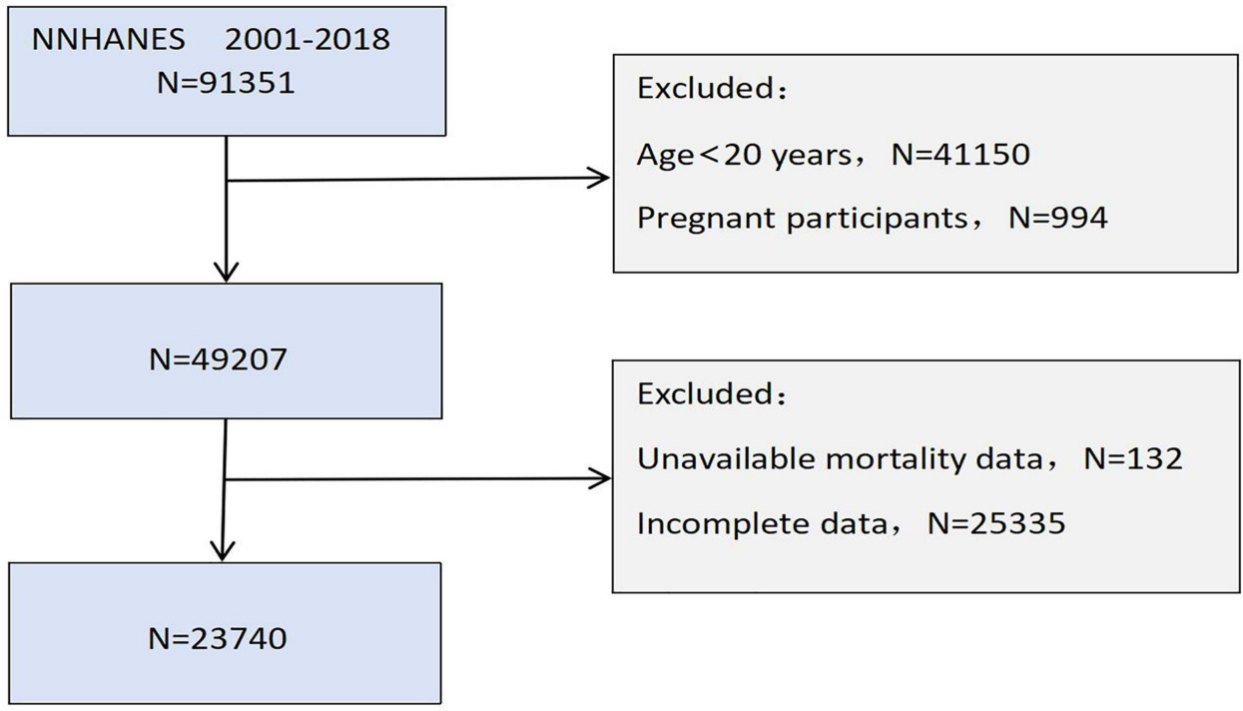
Flowchart of participants selection.

This investigation employed information from the National Health and Nutrition Examination Survey (NHANES) database (https://www.cdc.gov/nchs/nhanes/index.htm). NHANES operates as a nationally representative, multi-stage investigation executed by the National Center for Health Statistics (NCHS) and the Centers for Disease Control and Prevention (CDC), established to evaluate nutritional and health conditions among different age populations in the United States. The NCHS Ethics Review Board sanctioned all NHANES research protocols, with all participants providing informed consent. Since NHANES data are publicly available and fully de-identified, studies involving secondary analyses of the NHANES database are considered exempt from additional ethical approval according to the policy of our Institutional Review Board. The research selected adults aged 20 years and above as eligible subjects. Participants were excluded based on: (1) missing data on monocyte count or serum albumin; (2) pregnancy; (3) missing data on CVD; (4) absence of mortality data or loss to follow-up; and (5) missing key covariate information. Of the 91,351 individuals registered in NHANES between 2001 and 2018, 23,740 met the criteria for final analysis. The participant selection process is depicted in Figure 1.

### 2.2 Definition of MAR and CVD

The exposure variable, MAR, was derived from the complete blood cell count and standard biochemical parameters available in the NHANES database. Given the relatively small values and wide distribution range of MAR, and to mitigate the impact of extreme values on effect size, MAR was standardized in this study, each unit increase in standardized MAR corresponds to a change of one standard deviation in MAR. Furthermore, to better illustrate the impact of MAR on the occurrence and progression of CVD at different classification levels, this study categorized all participants’ MAR values into tertiles based on the distribution of the overall data: Tertile 1 (MAR < 0.10), Tertile 2 (0.10 ≤ MAR < 0.14), and Tertile 3 (MAR ≥ 0.14). Additionally, five composite inflammation indices, known to significantly influence disease outcomes, were included as covariates for comparative analysis: the Advanced Lung Cancer Inflammation Index (ALI), Neutrophil-to-Albumin Ratio (NAR), Hemoglobin, Albumin, Lymphocyte, and Platelet score (HALP), SII, and NLR (19–23). The calculation formulas for all indices are provided in the supplementary materials, Supplementary Table S1.

CVD diagnosis was established through participants’ self-disclosed medical records indicating congestive heart failure (CHF), coronary heart disease (CHD), angina, myocardial infarction, or stroke (24). The participants indicated whether healthcare providers or physicians had previously notified them about any of these medical conditions, with positive responses confirming CVD presence.

MAR integrates the biological mechanisms of monocytes and albumin, reflecting the pathophysiological balance between pro-inflammatory activity, nutritional status, and anti-inflammatory defense. Monocytes play a pivotal role in the initiation and progression of atherosclerosis through endothelial infiltration and cytokine release, representing a central mechanism in the pathogenesis of CVD (12). In contrast, serum albumin exerts protective effects by mitigating oxidative stress, exerting anti-inflammatory and antithrombotic functions, and maintaining cardiovascular homeostasis (13–16). Therefore, an elevated MAR indicates enhanced pro-inflammatory activity accompanied by diminished protective capacity, establishing a strong biological link with the development of CVD.

### 2.3 Assessment of mortality

Mortality data, including all-cause and cardiovascular mortality, were procured from the NDI public mortality dataset, linked with participants’ health data from NHANES 2001–2018. All-cause mortality encompassed deaths resulting from any cause, whereas cardiovascular mortality was identified per the International Classification of Diseases codes, including fatalities associated with heart disease (I00–I09, I11, I13, I20–I51) and cerebrovascular disease (I60–I69) (25). The follow-up period in person-years was calculated as the interval from the date of NHANES Mobile Examination Center (MEC) examination until either the date of mortality or the end of surveillance (December 31, 2019), whichever happened first.

### 2.4 Covariates

Based on relevant studies and considering potential confounders, this study incorporated the following covariates from NHANES interview and laboratory data: age (years); sex (male or female); race/ethnicity (non-Hispanic White, non-Hispanic Black, Mexican American, and other); educational background (less than high school, high school graduate, and post-high school); relationship status (married/Living with partner, widowed/divorced/separated/never married); family poverty income ratio (PIR) (≤ 1.30, 1.31–3.50, >3.50); total cholesterol level (TC, mg/dl); smoking habits (never smokers: persons who smoked fewer than 100 cigarettes throughout their lifetime; current smokers: individuals who smoked more than 100 cigarettes and continued smoking during the survey period; former smokers: subjects who smoked more than 100 cigarettes but stopped before the survey); alcohol intake (characterized as consuming more than one 12-ounce beer, one 5-ounce glass of wine, or 1.5 ounces of liquor in the previous year); body mass index (BMI): normal weight (<25 kg/m^2^), overweight (25–30 kg/m^2^), and obese (>30 kg/m^2^); diabetes (identified through self-reported history, antidiabetic medication or insulin usage, fasting plasma glucose ≥7.0 mmol/L, or hemoglobin A1c ≥ 6.5%) (24); hypertension (determined by the average of four measurements with systolic blood pressure ≥140 mmHg and/or diastolic blood pressure ≥90 mmHg) (26). Cancer, COPD, and CVDs were established through self-reported physician diagnoses. Additional information appears on the NHANES website.

### 2.5 Statistical analyses

Statistical analysis was performed following the guidelines set by the CDC, taking into consideration the intricate, multi-stage cluster survey design. Specific NHANES sample weightings were incorporated to ensure sample representativeness. For data with normal distribution, continuous measurements are displayed as mean ± standard deviation (SD), whereas non-normal continuous measurements are shown as median (interquartile range, IQR). Categorical data elements are expressed as counts (n) and proportions (%). The analysis of variance (ANOVA) method evaluated differences among continuous measurements, while chi-square testing assessed between-group variations for categorical elements.

After standardizing MAR, a multi-stage modeling approach was employed to explore the relationship between MAR and the risk of CVD and mortality. For the CVD investigation, a three-tiered multivariable logistic regression model was established: Model 1 remained unadjusted; Model 2 adjusted for demographic parameters (age, sex, and race); while Model 3 incorporated additional adjustments for potential confounding factors. The nonlinear relationships between MAR and CVD were investigated utilizing restricted cubic spline (RCS), while threshold effects were identified through likelihood ratio testing. Subgroup analyses per the demographic and clinical characteristics were conducted to evaluate effect modification. Furthermore, receiver operating characteristic (ROC) curve evaluation measured the diagnostic performance of MAR regarding various cardiovascular endpoints (CVD, CHF, CHD, angina, myocardial infarction, and stroke), comparing variations in areas under the curve (AUCs).

The mortality evaluation employed Cox proportional hazards modeling, incorporating an identical three-tiered adjustment approach to investigate associations between MAR and mortality endpoints, encompassing both all-cause and cardiovascular outcomes. The survival differences across groups utilized Kaplan–Meier curve analysis (categorized by MAR tertiles) alongside log-rank testing methods. To illustrate the dose-response correlation between MAR and death risk, RCS regression techniques were employed.

Multiple verification steps assessed result stability: (1) stratified examinations and effect modification testing utilizing principal covariates; (2) the main analyses were repeated according to quartiles of MAR: Quartile 1 (MAR < 0.10), Quartile 2 (0.10 ≤ MAR < 0.12), Quartile 3 (0.12 ≤ MAR < 0.16) and Quartile 4 (MAR ≥ 0.16); (3) additional sensitivity evaluations incorporating CVD condition and integrated inflammatory nutrition indicators; (4) To minimize the potential reverse causation bias, participants who died within 2 years of follow-up were excluded.

To quantify the significant predictive value of MAR for CVD and mortality outcomes, we constructed a baseline model that included age, gender, hypertension, diabetes, and composite inflammatory markers (NLR, NAR, ALI, SII, HALP). An extended model was then developed by incorporating the MAR indicator into the baseline model. A net reclassification improvement (NRI) analysis was performed to evaluate the improvement in predictive performance. Risk was classified as low or high based on the predicted probabilities from both models, with a threshold of 0.5. The statistical computations were conducted using the R 4.3.2 platform, with significance thresholds established at a two-tailed *P* value < 0.05.

## 3 Results

### 3.1 Baseline characteristics

Among the 23,740 participants in this investigation, 54.28% were male, and 45.72% were female, with a mean age of 46.94 ± 17.10 years. Of these, 49.09% were non-Hispanic White, and over half had completed at least high school education. A total of 1,920 participants had a diagnosis of CVD. Following stratification by MAR tertiles, a higher proportion of individuals with CVD, CHF, CHD, angina, myocardial infarction, and stroke were detected in the higher MAR tertiles. Characteristics more prevalent in the higher MAR tertiles included male sex, advanced age, smoking, obesity, lower income and education levels, and comorbidities like hypertension, diabetes, cancer, and COPD. Statistically significant baseline differences were detected between groups (*P* < 0.05), as demonstrated in Table 1.

**Table 1:** Baseline characteristics of study participants.

Characteristics	Total	Tertile 1 (<0.10)	Tertile 2 (0.10–0.14)	Tertile 3 (≥0.14)	*P*-value
*N*	23,740	7,616	7,771	8,353	
Age (years)	46.94 ± 17.10	45.39 ± 16.12	46.35 ± 16.94	48.89 ± 17.92	<0.001
Gender, *n* (%)					<0.001
Male	12,887 (54.28%)	3,700 (48.58%)	4,300 (55.33%)	4,887 (58.51%)	
Female	10,853 (45.72%)	3,916 (51.42%)	3,471 (44.67%)	3,466 (41.49%)	
Race, *n* (%)					<0.001
Mexican American	3,744 (15.77%)	1,244 (16.33%)	1,273 (16.38%)	1,227 (14.69%)	
Non-Hispanic White	11,654 (49.09%)	3,228 (42.38%)	3,974 (51.14%)	4,452 (53.30%)	
Non-Hispanic Black	4,492 (18.92%)	1,753 (23.02%)	1,289 (16.59%)	1,450 (17.36%)	
Other	3,850 (16.22%)	1,391 (18.26%)	1,235 (15.89%)	1,224 (14.65%)	
Education level, *n* (%)					<0.001
Less than high school	4,622 (19.47%)	1,386 (18.20%)	1,474 (18.97%)	1,762 (21.09%)	
High school or GED	5,383 (22.67%)	1,546 (20.30%)	1,725 (22.20%)	2,112 (25.28%)	
Above high school	13,735 (57.86%)	4,684 (61.50%)	4,572 (58.83%)	4,479 (53.62%)	
Marital status, *n* (%)					<0.001
Married/living with partner	14,541 (61.25%)	4,739 (62.22%)	4,847 (62.37%)	4,955 (59.32%)	
Widowed/divorced/separated/never married	9,199 (38.75%)	2,877 (37.78%)	2,924 (37.63%)	3,398 (40.68%)	
PIR, *n* (%)					<0.001
Low income (≤ 1.30)	6,110 (25.74%)	1,809 (23.75%)	1,955 (25.16%)	2,346 (28.09%)	
Medium income (1.31–3.50)	8,811 (37.11%)	2,793 (36.67%)	2,829 (36.40%)	3,189 (38.18%)	
High income (> 3.5）	8,819 (37.15%)	3,014 (39.57%)	2,987 (38.44%)	2,818 (33.74%)	
Smoking status, *n* (%)					<0.001
Never	11,818 (49.78%)	4,319 (56.71%)	3,945 (50.77%)	3,554 (42.55%)	
Former	7,251 (30.54%)	2,150 (28.23%)	2,425 (31.21%)	2,676 (32.04%)	
Current	4,671 (19.68%)	1,147 (15.06%)	1,401 (18.03%)	2,123 (25.42%)	
Alcohol use, *n* (%)					<0.001
Yes	15,376 (64.77%)	2,872 (37.71%)	2,693 (34.65%)	2,799 (33.51%)	
No	8,364 (35.23%)	4,744 (62.29%)	5,078 (65.35%)	5,554 (66.49%)	
BMI					<0.001
Normal	7,225 (30.43%)	2,781 (36.52%)	2,390 (30.76%)	2,054 (24.59%)	
Overweight	8,182 (34.47%)	2,628 (34.51%)	2,804 (36.08%)	2,750 (32.92%)	
Obese	8,333 (35.10%)	2,207 (28.98%)	2,577 (33.16%)	3,549 (42.49%)	
Hypertension, *n* (%)					<0.001
Yes	9,058 (38.16%)	2,533 (33.26%)	2,841 (36.56%)	3,684 (44.10%)	
No	14,682 (61.84%)	5,083 (66.74%)	4,930 (63.44%)	4,669 (55.90%)	
DM, *n* (%)					<0.001
Yes	3,070 (12.93%)	826 (10.85%)	868 (11.17%)	1,376 (16.47%)	
No	20,670 (87.07%)	6,790 (89.15%)	6,903 (88.83%)	6,977 (83.53%)	
CVD, *n* (%)					<0.001
Yes	1,920 (8.09%)	426 (5.59%)	544 (7.00%)	950 (11.37%)	
No	21,820 (91.91%)	7,190 (94.41%)	7,227 (93.00%)	7,403 (88.63%)	
Angina, *n* (%)					<0.001
Yes	505 (2.13%)	114 (1.50%)	145 (1.87%)	246 (2.95%)	
No	23,235 (97.87%)	7,502 (98.50%)	7,626 (98.13%)	8,107 (97.05%)	
Heart attack, *n* (%)					<0.001
Yes	752 (3.17%)	144 (1.89%)	227 (2.92%)	381 (4.56%)	
No	22,988 (96.83%)	7,472 (98.11%)	7,544 (97.08%)	7,972 (95.44%)	
CHF, *n* (%)					<0.001
Yes	502 (2.11%)	88 (1.16%)	127 (1.63%)	287 (3.44%)	
No	23,238 (97.89%)	7,528 (98.84%)	7,644 (98.37%)	8,066 (96.56%)	
CHD, *n* (%)					<0.001
Yes	797 (3.36%)	152 (2.00%)	221 (2.84%)	424 (5.08%)	
No	22,943 (96.64%)	7,464 (98.00%)	7,550 (97.16%)	7,929 (94.92%)	
Stroke, *n* (%)					<0.001
Yes	576 (2.43%)	143 (1.88%)	163 (2.10%)	270 (3.23%)	
No	23,164 (97.57%)	7,473 (98.12%)	7,608 (97.90%)	8,083 (96.77%)	
COPD, *n* (%)					<0.001
Yes	377 (1.59%)	80 (1.05%)	97 (1.25%)	200 (2.39%)	
No	23,363 (98.41%)	7,536 (98.95%)	7,674 (98.75%)	8,153 (97.61%)	
Cancer, *n* (%)					<0.001
Yes	2,044 (8.61%)	547 (7.18%)	656 (8.44%)	841 (10.07%)	
No	21,696 (91.39%)	7,069 (92.82%)	7,115 (91.56%)	7,512 (89.93%)	
MAR	0.13 ± 0.05	0.09 ± 0.01	0.12 ± 0.01	0.18 ± 0.05	<0.001
Monocyte	0.56 ± 0.20	0.37 ± 0.07	0.53 ± 0.05	0.75 ± 0.19	<0.001
Albumin	4.26 ± 0.33	4.33 ± 0.33	4.31 ± 0.30	4.16 ± 0.33	<0.001
NLR	2.15 ± 1.13	2.02 ± 1.08	2.09 ± 0.96	2.33 ± 1.28	<0.001
NAR	1.00 ± 0.42	0.82 ± 0.33	0.95 ± 0.33	1.21 ± 0.49	<0.001
ALI	69.83 ± 48.23	72.20 ± 39.06	70.07 ± 36.25	67.44 ± 63.15	<0.001
HALP	0.56 ± 0.66	0.51 ± 0.22	0.56 ± 0.26	0.61 ± 1.06	<0.001
SII	541.44 ± 326.60	491.40 ± 297.48	518.73 ± 276.62	608.19 ± 379.60	<0.001
TC	5.05 ± 1.07	5.09 ± 1.06	5.08 ± 1.06	4.98 ± 1.08	<0.001

MAR, monocyte-albumin ratio; NLR, neutrophil-lymphocyte ratio; NAR, neutrophil-albumin ratio; ALI, advanced lung cancer inflammation index; HALP, Hemoglobin, albumin, lymphocyte and platelet scores; SII, systemic immune inflammation index; TC, total Cholesterol; PIR, poverty income ratio; BMI, body mass index; DM, diabetes; CVD, cardiovascular disease; CHF, congestive heart-failure; CHD, coronary heart disease.

### 3.2 Link between MAR and CVD

Table 2 demonstrates the findings from a multivariable logistic regression analysis investigating the link between MAR and CVD across three distinct models. The investigation identified a substantial positive link between MAR and CVD. After standardizing MAR, the unadjusted Model 1 indicated that for each single-unit elevation in MAR, there was a 1.36-unit enhancement in CVD risk (95% CI: 1.26–1.47, *P* < 0.001). After adjusting for confounders in Models 2 and 3, the association weakened, although it remained significant in both adjusted models. Within the completely adjusted Model 3, every single-unit increment in MAR correlated with a 1.09-fold rise in CVD occurrence (95% CI: 1.03–1.16, *P* = 0.004), indicating that MAR remains significantly associated with CVD after accounting for confounding factors. Upon stratification of MAR into tertiles, subjects in the highest tertile exhibited a 35% elevated probability of CVD development vs. individuals in the lowest tertile (95% CI: 1.13–1.61, *P* = 0.001). Trend tests using MAR as a continuous variable revealed a notable increasing trend in CVD risk across all three models (*P* < 0.05), suggesting a statistically significant rise in CVD risk as MAR levels increase.

**Table 2:** Association between MAR and other inflammatory biomarkers with CVD.

Variables	Model 1^c^		Model 2^d^		Model 3^e^	
	^a^OR (95%CI^b^)	*P*-value	OR (95%CI)	*P*-value	OR (95%CI)	*P*-value
MAR	1.36 (1.26–1.47)	<0.001	1.25 (1.16–1.34)	<0.001	1.09 (1.03–1.16)	0.004
Tertile 1	1.00 (Reference)		1.00 (Reference)		1.00 (Reference)	
Tertile 2	1.41 (1.19–1.67)	<0.001	1.29 (1.09–1.54)	0.004	1.19 (0.98–1.44)	0.080
Tertile 3	2.30 (1.96–2.70)	<0.001	1.85 (1.57–2.17)	<0.001	1.35 (1.13–1.61)	0.001
*P* for trend	<0.001		<0.001		0.002	
NLR	1.23 (1.18–1.29)	<0.001	1.12 (1.06–1.17)	<0.001	1.04 (0.98–1.09)	0.218
Tertile 1	1.00 (Reference)		1.00 (Reference)		1.00 (Reference)	
Tertile 2	1.11 (0.95–1.30)	0.186	1.06 (0.89–1.26)	0.525	0.99 (0.83–1.19)	0.914
Tertile 3	1.83 (1.53–2.18)	<0.001	1.40 (1.16–1.69)	<0.001	1.12 (0.92–1.37)	0.262
*P* for trend	<.001		<.001		0.160	
NAR	1.47 (1.29–1.69)	<0.001	1.68 (1.44–1.96)	<0.001	1.05 (0.90–1.23)	0.524
Tertile 1	1.00 (Reference)		1.00 (Reference)		1.00 (Reference)	
Tertile 2	1.17 (0.99–1.39)	0.061	1.15 (0.97–1.37)	0.107	0.98 (0.82–1.17)	0.817
Tertile 3	1.58 (1.34–1.85)	<0.001	1.66 (1.41–1.97)	<0.001	1.05 (0.88–1.25)	0.593
*P* for trend	<.001		<.001		0.523	
ALI	1.00 (1.00–1.00)	0.394	1.00 (1.00–1.00)	0.703	1.00 (1.00–1.00)	0.934
Tertile 1	1.00 (Reference)		1.00 (Reference)		1.00 (Reference)	
Tertile 2	0.71 (0.60–0.83)	<0.001	0.88 (0.75–1.04)	0.137	0.91 (0.76–1.09)	0.303
Tertile 3	0.70 (0.60–0.81)	<0.001	0.89 (0.75–1.06)	0.193	0.87 (0.72–1.05)	0.155
*P* for trend	<.001		0.328		0.144	
SII	1.01 (1.01–1.01)	0.008	1.00 (1.00–1.00)	0.079	1.00 (1.00–1.00)	0.459
Tertile 1	1.00 (Reference)		1.00 (Reference)		1.00 (Reference)	
Tertile 2	0.84 (0.73–0.97)	0.018	0.84 (0.72–0.98)	0.025	0.77 (0.66–0.90)	0.001
Tertile 3	1.01 (0.86–1.18)	0.910	0.97 (0.83–1.15)	0.748	0.80 (0.67–0.96)	0.019
*P* for trend	0.658		0.851		0.058	
HALP	1.13 (1.02–1.25)	0.026	1.11 (0.99–1.25)	0.078	1.07 (0.99–1.16)	0.075
Tertile 1	1.00 (Reference)		1.00 (Reference)		1.00 (Reference)	
Tertile 2	0.92 (0.79–1.07)	0.288	1.03 (0.87–1.21)	0.736	1.06 (0.89–1.27)	0.506
Tertile 3	1.01 (0.87–1.17)	0.894	1.25 (1.07–1.46)	0.006	1.16 (0.99–1.37)	0.071
*P* for trend	0.790		0.007		0.082	

In the correlation analysis, MAR was standardized, and MAR, NLR, NAR, ALI, SII, and HALP were categorized into tertiles for further analysis.

^a^ OR, odds ratio.

^b^ 95% Cl, 95% confidence interval.

^c^ Model 1: No covariates were adjusted.

^d^ Model 2: Adjusted for age, sex, and race.

^e^ Model 3: Adjusted for age, sex, race, marital status, education level, BMI, PIR, TC, alcohol use, smoking status, hypertension, DM, COPD, and cancer.

**Figure 2:**
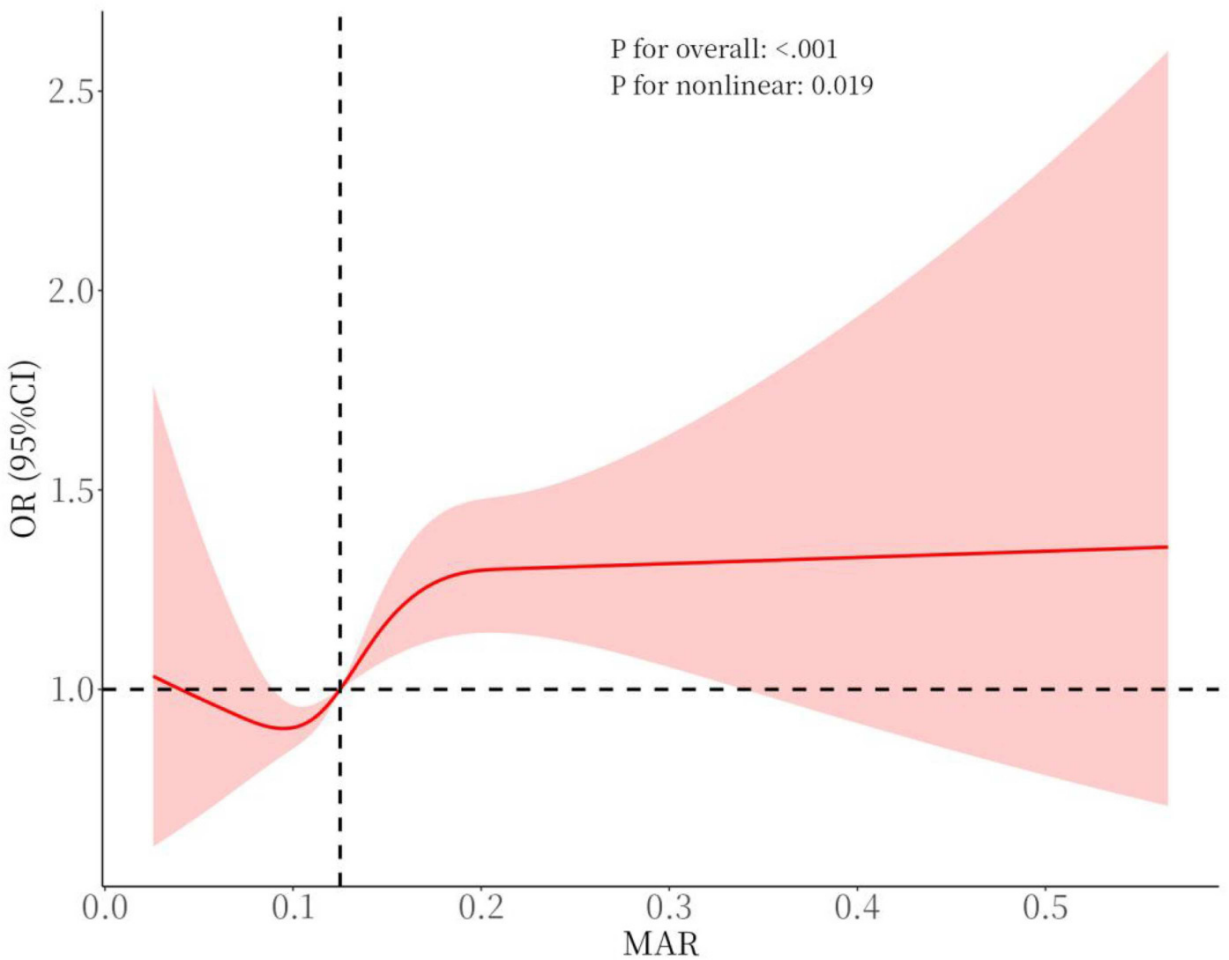
Smooth curve fitting for MAR with CVD.

For other composite inflammatory markers, the unadjusted model revealed significant associations with CVD risk for all markers except ALI. In the crude model, the second and third tertiles of ALI were significantly correlated with CVD. After adjusting for confounders, only SII in the second and third tertiles remained associated with CVD, indicating that the relationship between SII and CVD may be primarily influenced by confounding factors. In contrast, the link between MAR and CVD exhibited stability, demonstrating a notable connection following comprehensive adjustment. Compared to other composite inflammatory markers, MAR showed a superior capacity to predict CVD risk. To explore the nonlinear correlation between MAR and CVD, an RCS curve was constructed, revealing a potential nonlinear association (Figure 2). However, threshold effect analysis did not identify a significant inflection point, suggesting the absence of a clear threshold effect between MAR and CVD. This may be due to the limited moderating effects of potential covariates or the restricted range of moderator variable values, which could obscure the manifestation of threshold effects (Table 3).

**Table 3:** Threshold effect analysis of MAR on CVD utilizing a two-piecewise linear regression model in model 3.

Outcome	Effect	*P*-value
Fitting model by standard linear regression	4.74 (1.93–11.62)	<0.001
Fitting model by two-piecewise linear regression		
Inflection point	0.17	
<0.07	28.59 (3.24–252.20)	0.003
≥0.07	1.43 (0.31–6.61)	0.643
*P* for likelihood test		0.054

### 3.3 Subgroup and sensitivity analysis between MAR and CVD

**Figure 3:**
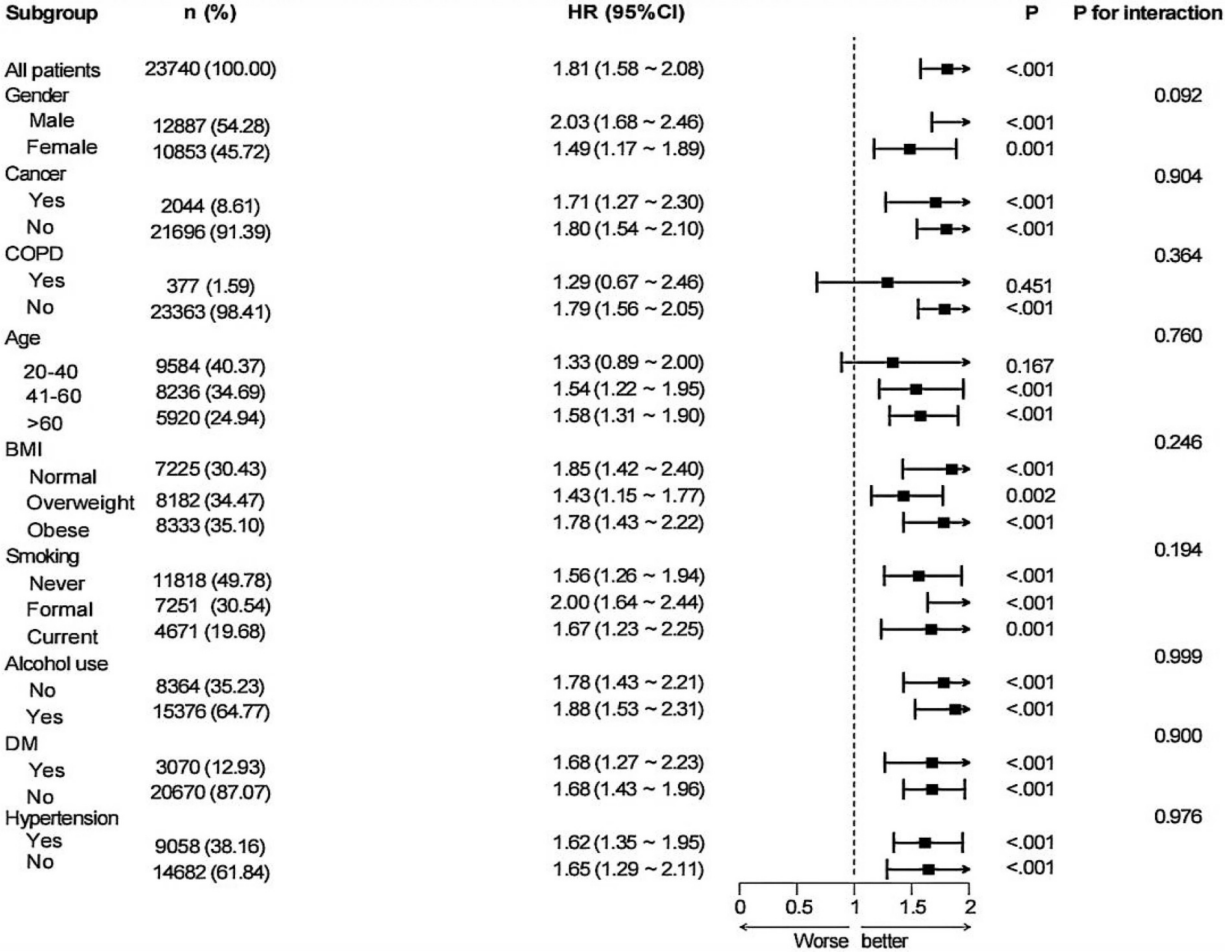
Subgroup analysis for the associations of MAR with CVD.

Subgroup analyses were performed based on nine variables—age, sex, BMI, cancer, COPD, alcohol consumption, hypertension, diabetes, and smoking status—to examine the robustness and possible variability of the link between MAR and CVD across different demographic and clinical subpopulations (Figure 3). A notable correlation between MAR and CVD remained evident across all categories, excluding patients with COPD and participants aged 20–40 years (*P* < 0.05). The findings indicate that the link between MAR and CVD exhibits stronger significance among participants aged above 40 years. Conversely, the relationship appears weaker or insignificant in those with COPD, likely due to confounding factors associated with the disease. Interaction analyses suggested no significant interaction effects between the eight subgroup factors and the MAR-CVD association.

The results (Supplementary Table S2) of the sensitivity analysis based on MAR quartile grouping showed that, in all three models, as MAR increased, the risk of CVD progressively elevated after reaching a certain threshold. In Model 3, the odds ratios (OR) for the third and fourth quartiles were 1.25 (95% CI: 1.01–1.54) and 1.39 (95% CI: 1.12–1.73), respectively (*P* for both < 0.05). The trend test yielded a *P*-value of < 0.05. This indicates that after stratification by quartiles, the relationship between MAR and CVD remains consistent, further confirming the stability of the results in this study.

### 3.4 ROC analysis

**Figure 4:**
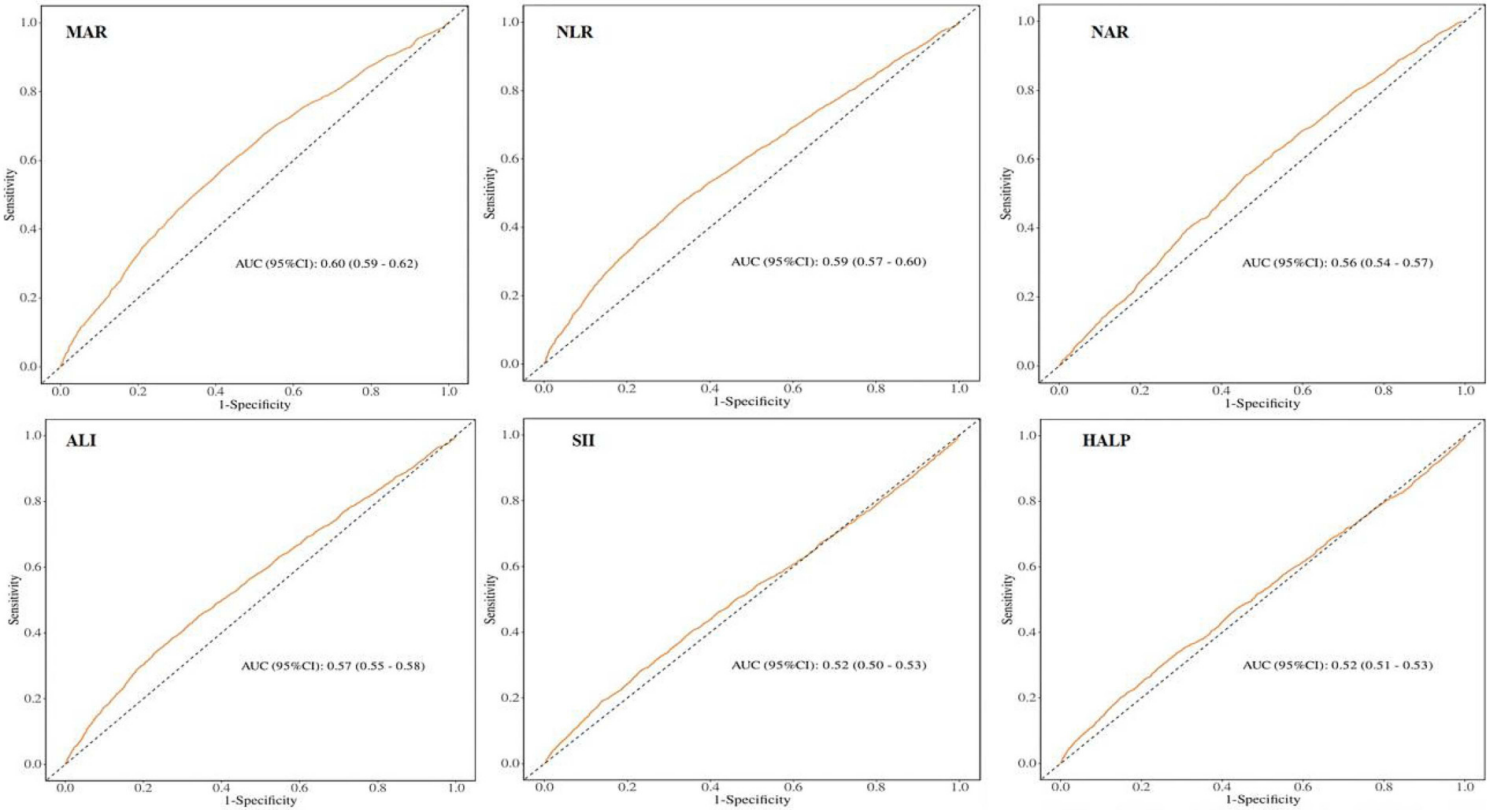
ROC curves and the AUC values of the six inflammatory biomarkers in diagnosing CVD.

**Figure 5:**
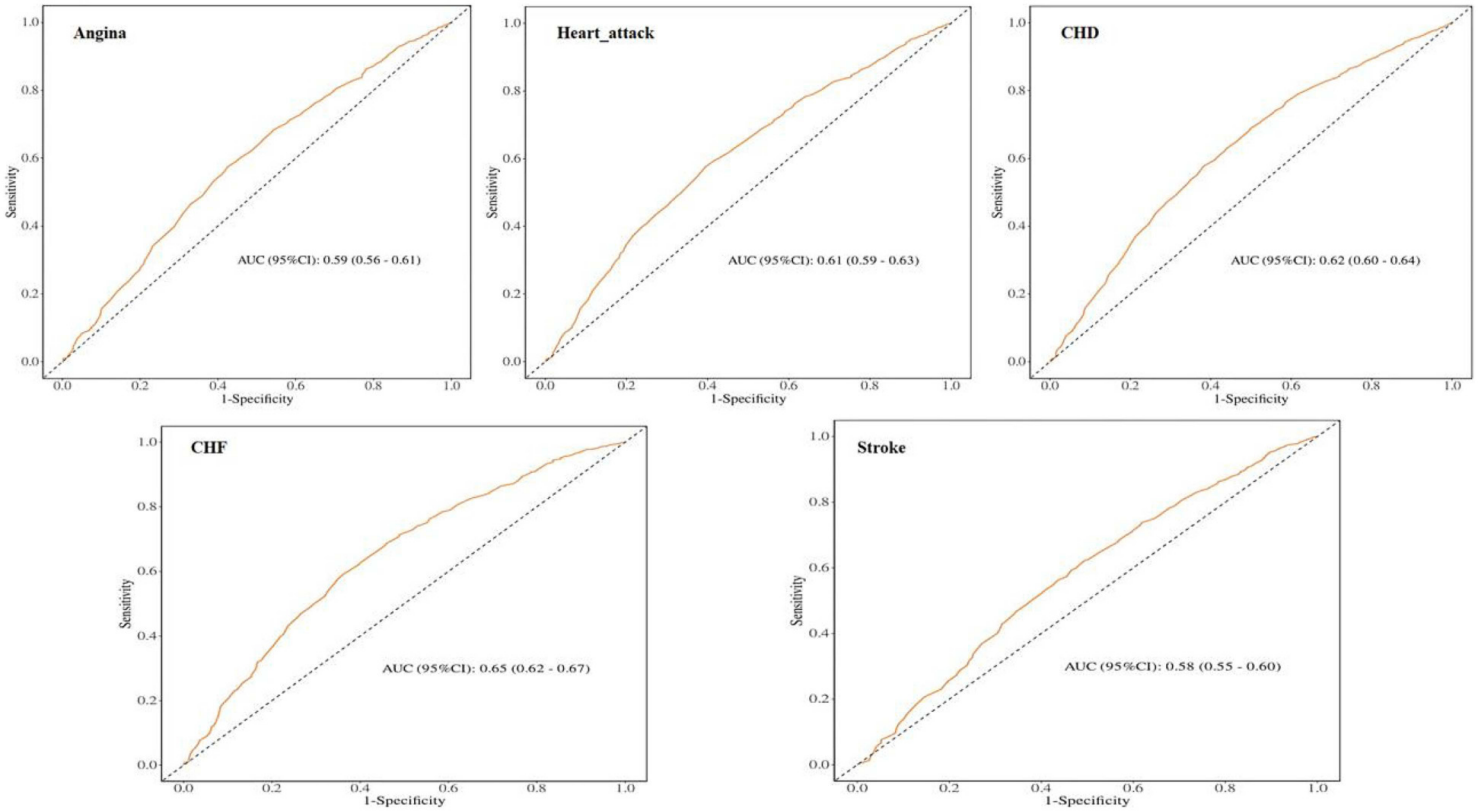
ROC curves and the AUC values of MAR in diagnosing CVD events.

To evaluate the prognostic performance of MAR vs. other composite inflammatory markers (encompassing NLR, NAR, ALI, SII, and HALP) for CVD, the AUC was calculated, as shown in Figure 4. The results confirm that MAR exhibits considerable predictive value relative to other inflammatory biomarkers. Additionally, ROC curve analyses of MAR for angina, myocardial infarction, CHD, CHF, and stroke are presented in Figure 5. Among these outcomes, CHF demonstrated the highest AUC, followed by CHD, suggesting that MAR may offer superior discriminatory power for predicting CHF and CHD compared to other CVD outcomes. This may indicate a stronger association between MAR and CHF or CHD or a more prominent role of MAR in their pathophysiological mechanisms. The NRI analysis showed that the addition of MAR significantly improved risk reclassification. The NRI value was 0.9, suggesting that the inclusion of MAR enhanced the ability to correctly reclassify individuals into higher or lower risk groups.

### 3.5 Association of MAR with mortality

**Figure 6:**
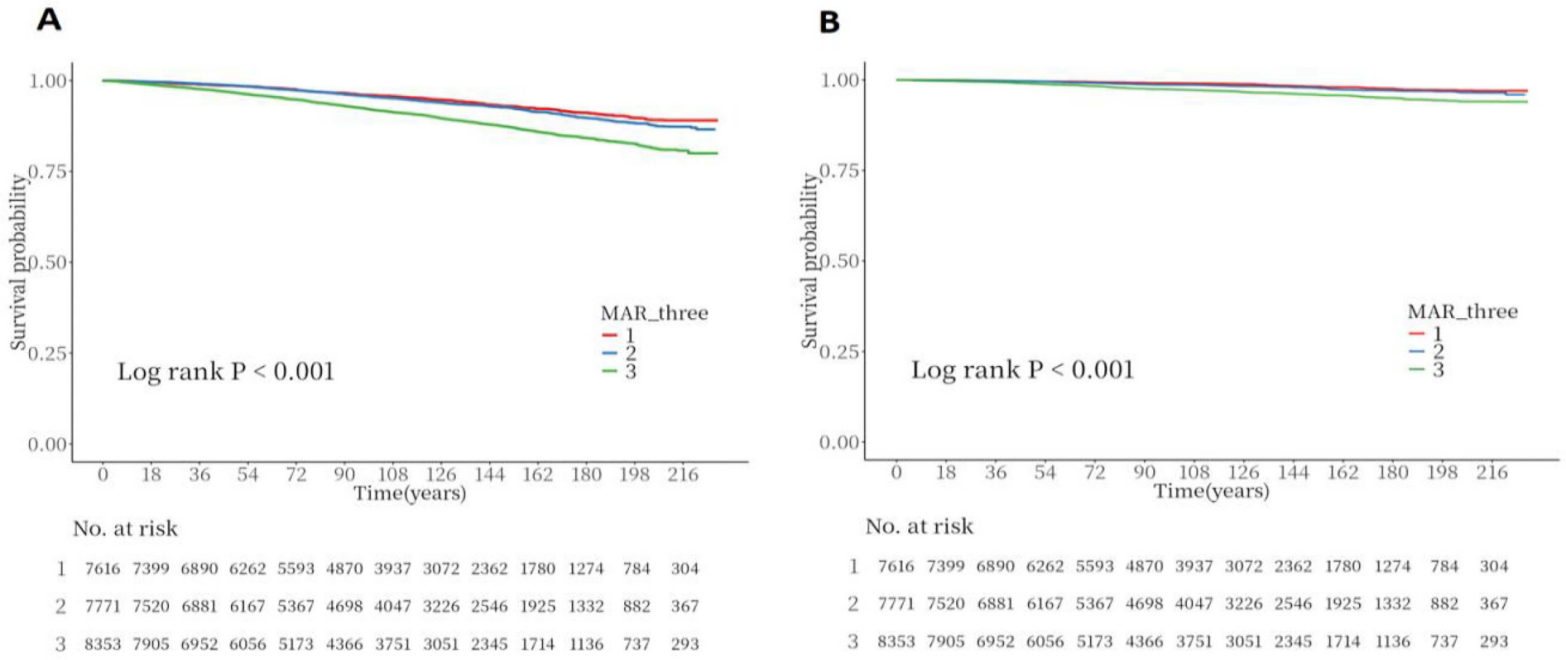
Kaplan–meier survival analysis curves for all-cause and CVD-cause mortality. **(A)** Kaplan–Meier analysis for all-cause mortality; **(B)** Kaplan–Meier analysis for CVD-cause mortality.

Throughout a median follow-up of 8.3 years (interquartile range: 4.9–13.0 years), the study documented 2,338 all-cause deaths and 705 CVD-related deaths. The link between MAR and mortality underwent examination via Cox regression analysis utilizing three sequential adjustment models. The MAR values were divided into tertile categories. Model 1 (unadjusted) demonstrated that subjects in the highest MAR tertile exhibited markedly increased risks for all-cause mortality [hazard ratio [HR]: 1.94, 95% confidence interval [CI]: 1.70–2.23] and CVD mortality (HR: 2.40, 95% CI: 1.84–3.12), with *P* < 0.001. After comprehensive covariate adjustment, Model 3 indicated that within the highest tertile, each incremental unit of MAR corresponded to a 25% heightened risk of all-cause mortality and a 46% elevated risk of CVD mortality (Table 4). The Kaplan–Meier analysis illustrated notably diminished survival probabilities for both all-cause and CVD mortality in participants within the highest MAR tertile vs. those in the lowest tertile (*P* < 0.001; Figures 6A,6B).

**Table 4:** Cox regression of the link between MAR and all-cause mortality and CVD-cause mortality in the general population.

Mortality type/Variable	Model 1^c^		Model 2^d^		Model 3^e^	
	^a^HR (95%CI^b^)	*P-*value	HR (95%CI)	*P-*value	HR (95%CI)	*P-*value
All-cause mortality						
Tertile 1	1.00 (Reference)		1.00 (Reference)		1.00 (Reference)	
Tertile 2	1.12 (0.96–1.32)	0.150	1.02 (0.86–1.20)	0.846	0.95 (0.80–1.12)	0.559
Tertile 3	1.94 (1.70–2.23)	<0.001	1.53 (1.33–1.76)	<0.001	1.25 (1.08–1.44)	0.002
*P* for trend		<0.001		<0.001		<0.001
Cardiovascular mortality						
Tertile 1	1.00 (Reference)		1.00 (Reference)		1.00 (Reference)	
Tertile 2	1.29 (0.97–1.72)	0.084	1.12 (0.83–1.49)	0.459	1.06 (0.79–1.43)	0.680
Tertile 3	2.40 (1.84–3.12)	<0.001	1.74 (1.31–2.30)	<0.001	1.46 (1.10–1.93)	0.008
*P* for trend		<0.001		<0.001		0.002

^a^ HR, hazard ratio.

^b^ 95% Cl, 95% confidence interval.

^c^ Model 1: No covariates were adjusted.

^d^ Model 2: Adjusted for age, sex, and race.

^e^ Model 3: Adjusted for age, sex, race, marital status, education level, BMI, PIR, TC, alcohol use, smoking status, hypertension, DM, COPD, and cancer.

### 3.6 Nonlinear regression of MAR and mortality

**Figure 7:**
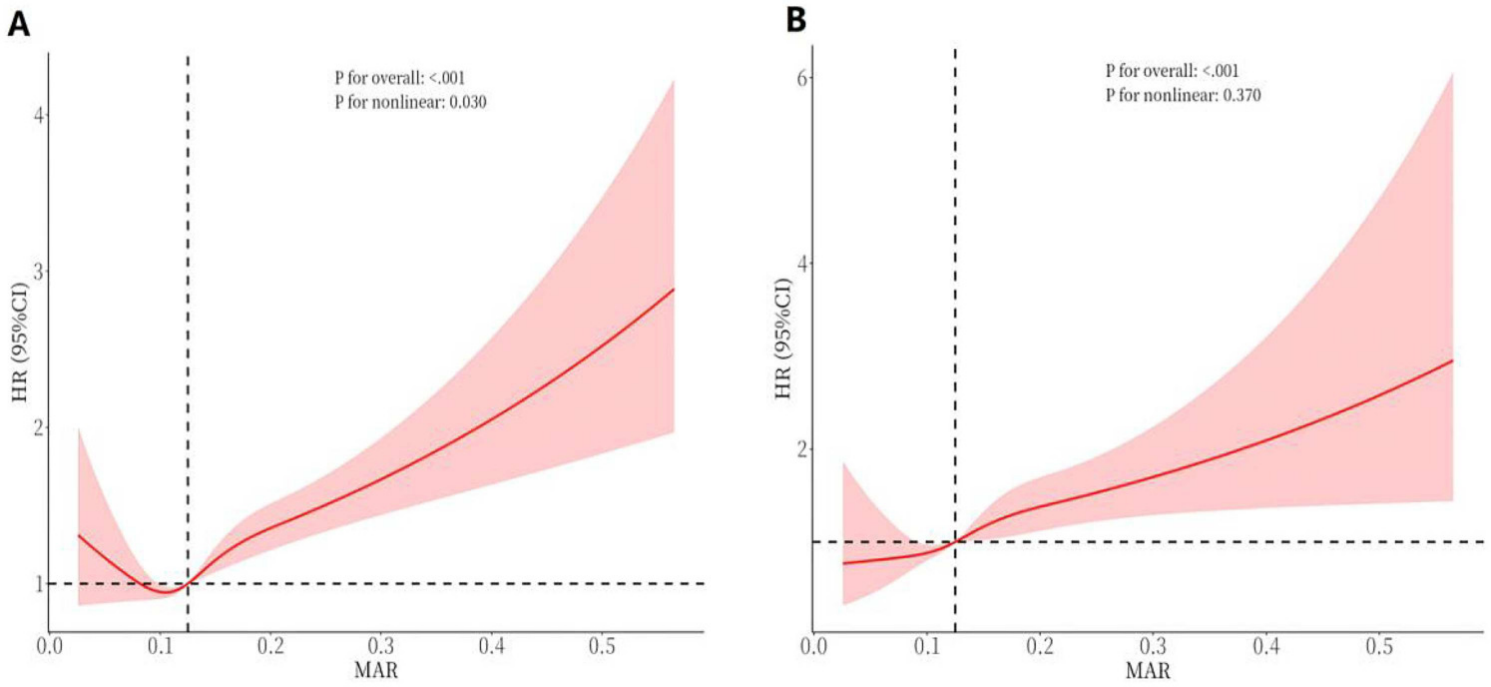
RCS curves of MAR impact on long-term ALL-cause and CVD-cause mortality in the general population. **(A)** All-cause mortality; **(B)** CVD-cause mortality.

The RCS curve depicted in Figure 7A shows a nonlinear connection between continuous MAR levels and all-cause mortality (*P* for nonlinearity = 0.030). Specifically, lower MAR values were linked to a reduced mortality risk, but once MAR exceeded 0.125, the all-cause mortality risk showed progressive elevation. The RCS analysis in Figure 7B indicates an ascending pattern in cardiovascular mortality risk with elevating MAR levels, with the link between MAR and cardiovascular mortality appearing either linear or without significant nonlinearity (*P* for nonlinearity = 0.370).

### 3.7 Subgroup and sensitivity analysis between MAR and mortality rate

**Figure 8:**
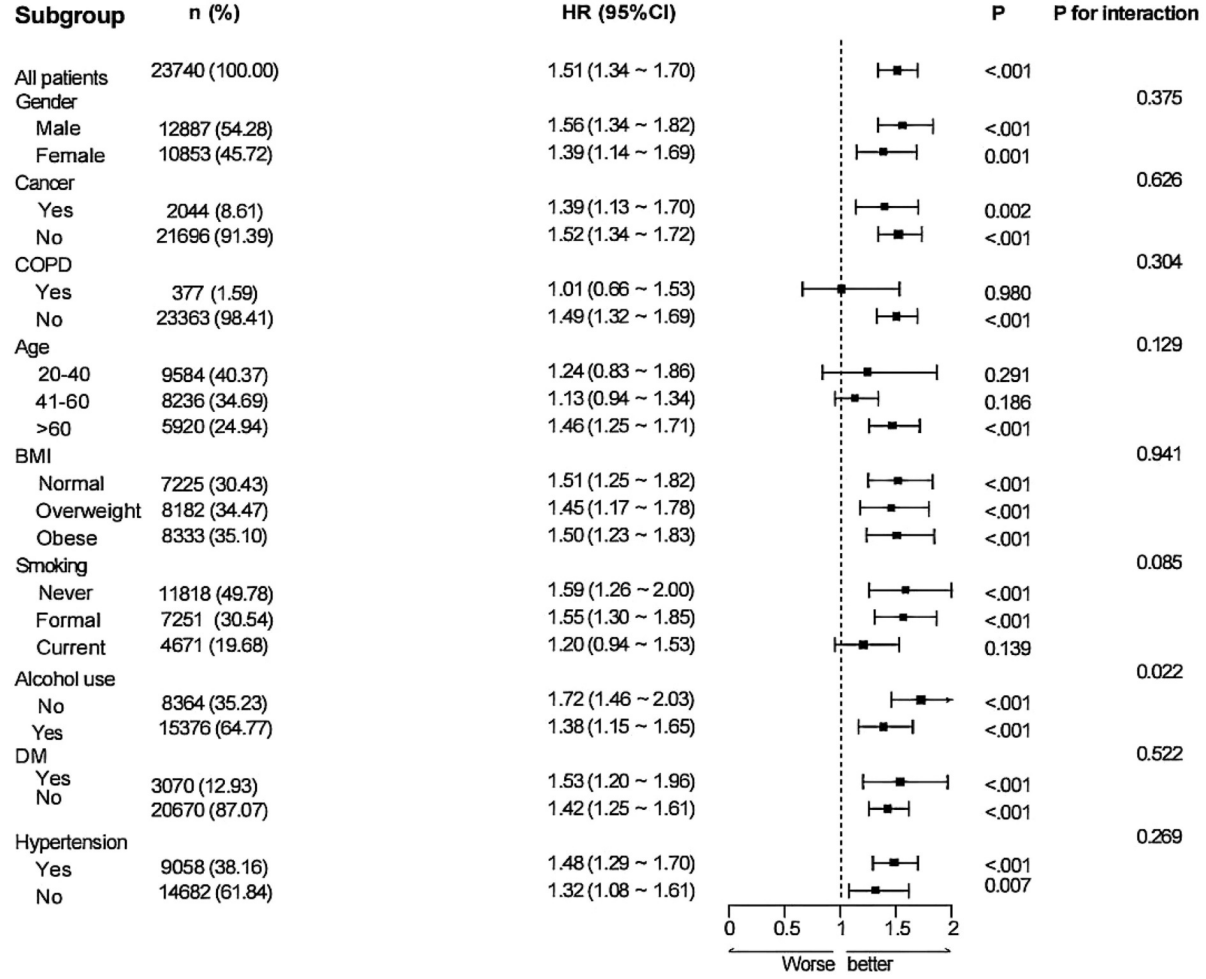
Subgroup analysis of the links between MAR and ALL-cause mortality in general population.

**Figure 9:**
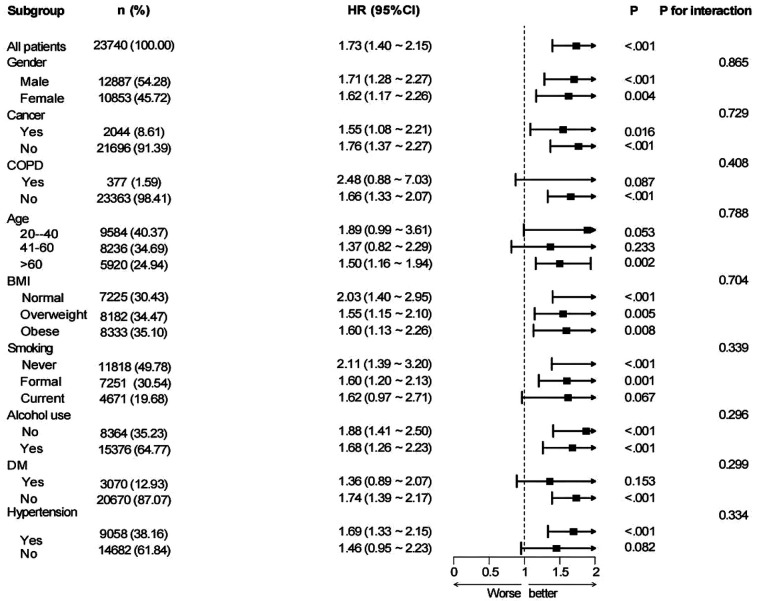
Subgroup analysis of the links between MAR and CVD-cause mortality in general population.

Subgroup analysis stratified by nine factors—age, sex, BMI, cancer, COPD, alcohol consumption, hypertension, diabetes, and smoking status—indicated a positive link between higher MAR and elevated all-cause mortality risk in the subgroups defined by sex, BMI, cancer, alcohol consumption, diabetes, and hypertension. This association was particularly pronounced in individuals aged ≥60 years. No notable association was detected among smokers or those with COPD (*P* > 0.05). Interaction analysis identified a potential interaction between alcohol consumption and MAR regarding all-cause mortality risk, suggesting that alcohol consumption may modulate the predictive value of MAR for mortality (*P* for interaction < 0.05). No notable interactions were detected between MAR and the other factors (*P* for interaction >0.05) (Figure 8). In the subgroup analysis of MAR and CVD mortality, no significant associations were observed among individuals with cancer, COPD, diabetes, current smoking status, or those without hypertension. Conversely, MAR demonstrated greater predictive accuracy for CVD mortality risk in individuals aged ≥60 years. No notable interactions between MAR and the aforementioned stratification factors were observed regarding CVD mortality (*P* for interaction > 0.05) (Figure 9).

The sensitivity analysis based on MAR quartile grouping (Supplementary Table **S3**) demonstrated a positive association between higher MAR levels and the risk of both all-cause mortality and CVD mortality in the general population (*P* for the fourth quartile < 0.05). These findings were consistent with those obtained from the analysis using MAR tertile grouping, indicating that elevated MAR levels are associated with an increased risk of mortality outcomes. Furthermore, the sensitivity analysis results, adjusted for cardiovascular disease history (Supplementary Table S4) and other inflammatory composite markers (Supplementary Table S5), remained consistent, further reinforcing the positive association between elevated MAR levels and increased all-cause and cardiovascular mortality in the general population (*P*-values for the third quartile were all < 0.05). Additionally, after excluding deaths occurring within the first 2 years of follow-up, Cox regression analysis showed that the risks of both all-cause and cardiovascular mortality remained significantly higher in the third quartile of MAR (OR: 2.24, 95% CI: 1.69–2.97, *P* < 0.05), with results remaining consistent after adjustment for confounding factors (Supplementary Table S6). These sensitivity analyses confirm that the associations observed in this study are robust and consistent across different models.

Finally, the NRI analysis confirmed the incremental value of MAR in predicting mortality outcomes. Incorporating MAR into the baseline model significantly improved risk reclassification (NRI >0.5). These findings indicate that MAR provides additional predictive power beyond established risk factors and composite inflammatory markers.

## 4 Discussion

This is the first large-scale study to demonstrate a significant association between elevated MAR levels and the risks of CVD, all-cause mortality, and cardiovascular-specific mortality in the general population. Our findings revealed a nonlinear relationship between MAR and these outcomes, with stronger associations observed among older adults. The robustness of our findings was consistently supported by stratified and sensitivity analyses, indicating that elevated MAR levels reliably predict the risk of CVD and mortality. Moreover, ROC analysis indicated that MAR exhibited superior predictive performance for CVD compared with other established composite inflammatory indices, with particularly stronger discriminative ability for heart failure and CHD. NRI analysis further demonstrated that MAR provides incremental predictive value for both CVD and mortality outcomes. Collectively, these findings highlight MAR as a reliable biomarker for CVD, all-cause mortality, and cardiovascular mortality in the general population.

MAR, as a novel composite inflammation–nutrition index, integrates the pro-inflammatory activity of monocytes with the protective role of albumin, thereby reflecting the pathophysiological balance between inflammation and nutritional status. Research has demonstrated that inflammatory processes, both systemic and local, significantly influence CVD development, spanning from initial endothelial impairment to observable clinical symptom (27–29). Inflammatory biomarkers have been identified as predictive indicators of CVD (30, 31). Nutritional status likewise exerts a critical influence on the onset and prognosis of CVD. Malnutrition and inadequate energy–protein intake can impair antioxidant defenses and immune competence, thereby heightening susceptibility to inflammation and accelerating atherosclerosis and vascular injury (32). Conversely, adequate nutrition helps preserve metabolic homeostasis and vascular function, mitigates oxidative stress and chronic inflammation, and ultimately confers cardiovascular protection (33). Therefore, the balance between inflammation as a pathogenic driver and nutrition as a protective factor represents a fundamental determinant of cardiovascular health. Our findings further substantiate this mechanistic framework, demonstrating that elevated MAR, as an integrated indicator of inflammation and nutritional status, is closely associated with an increased risk of CVD and adverse outcomes. This can be attributed to the components that constitute MAR, which integrates the pro-inflammatory role of monocytes and the protective function of albumin. Monocytes, serving as essential elements of the innate immune system, facilitate atherosclerosis formation and advancement of cardiovascular incidents by mediating inflammatory responses (34–36). In contrast, serum albumin, while primarily an important nutritional marker, also possesses significant antioxidant properties, scavenging free radicals and playing a protective role in maintaining homeostasis and cardiovascular health (37). Low serum albumin levels reflect systemic inflammation and heightened infection susceptibility (38) and serve as independent predictors of atherosclerosis progression and myocardial infarction prognosis (39–41), while being strongly linked to both cardiovascular and all-cause mortality (42). Thus, MAR offers a unique composite indicator for cardiovascular risk assessment by concurrently reflecting two key pathophysiological processes: inflammation (via monocytes) and protection (via albumin). Our study confirms that elevated MAR levels signify an exacerbation of the inflammation-nutrition imbalance, which can predict the onset of CVD and adverse clinical outcomes.

The subgroup analyses in this study revealed that the association between MAR and the risks of CVD and mortality was more pronounced among older individuals and those with hypertension or diabetes, whereas the effect was relatively attenuated in younger populations. This pattern suggests that the pathophysiological impact of inflammation–nutrition imbalance is particularly amplified in elderly and metabolically compromised groups. Previous studies have shown that older adults are characterized by low-grade chronic inflammation and suboptimal nutritional status. This internal milieu not only heightens the susceptibility of the cardiovascular system to inflammatory insults but also diminishes the body's compensatory capacity, thereby increasing the risk of disease onset and progression (43). In contrast, younger individuals generally have a lower baseline risk and possess stronger metabolic and immune resilience, which may attenuate the observable associations and contribute to effect dilution (44). Similarly, in individuals with metabolic disorders such as hypertension or diabetes, elevated MAR not only mirrors intensified inflammatory activity but also signifies impairment of nutritional–metabolic homeostasis, thereby serving as a more sensitive and clinically informative indicator of risk (45, 46). Therefore, our findings demonstrate a consistent effect, indicating that MAR holds greater value for risk stratification among older adults and individuals with metabolic comorbidities. This underscores its potential utility in identifying susceptible populations and guiding timely preventive interventions. However, in populations with a high inflammatory background, such as patients with COPD or current smokers, the association between MAR and adverse outcomes appeared unstable. This phenomenon may be attributable to the elevated baseline inflammatory status, concomitant therapeutic interventions, and multiple comorbidities commonly observed in these populations (47), which could induce fluctuations in monocyte counts or serum albumin levels. Such variability may increase exposure misclassification and, consequently, attenuate the true effect estimates. This finding suggests that, in settings characterized by heightened inflammatory activity or the presence of multiple comorbidities, the predictive capacity of a single composite index such as MAR may be inherently limited. Under these conditions, more reliable risk stratification is likely to require an integrated approach that combines multidimensional markers of inflammation, nutritional status, and clinical characteristics.

Notably, our ROC analysis across CVD subtypes revealed that MAR exhibited markedly stronger discriminatory ability for heart failure and coronary heart disease, whereas its predictive performance for stroke was comparatively weaker. This heterogeneity may be attributable to the fact that heart failure and coronary heart disease are more profoundly driven by chronic low-grade inflammation and metabolic dysregulation (4). Moreover, the protein–energy wasting that often accompanies chronic illness can accelerate the decline in serum albumin levels. As such, MAR, which integrates both inflammatory and nutritional dimensions, may more directly capture these underlying pathophysiological mechanisms. In contrast, stroke is often driven by multiple acute risk factors, such as atrial fibrillation and acute vascular injury (48), this may attenuate the observed effect of MAR. This differential finding suggests that MAR may hold particular value for risk stratification in CVD subtypes predominantly driven by chronic inflammation and nutritional imbalance. In addition, when MAR was compared with other established composite inflammatory indices included in our study (NLR, SII, NAR, HALP, and ALI), all demonstrated significant associations with CVD in unadjusted models; however, after sequential adjustment for multiple confounders, only MAR retained statistical significance. ROC analyses based on different inflammatory indices further demonstrated that MAR exhibited superior predictive value for CVD compared with other markers. This discrepancy may be attributed to the unique pathophysiological foundation of MAR, which not only captures the pro-inflammatory activity mediated by monocytes but also integrates nutritional and anti-inflammatory defense information, thereby more comprehensively reflecting the pathological process of inflammation–nutrition imbalance. In contrast, most other composite indices typically represent only a single aspect of the inflammatory response (49), making their predictive effects more susceptible to attenuation after adjustment for multiple confounders. Moreover, our NRI analyses confirmed the incremental predictive value of MAR over these indices, further underscoring the robustness and stability of the associations observed in our study models.

Nevertheless, although this study systematically compared MAR with several established composite inflammatory indices (NLR, NAR, ALI, SII, and HALP) and further incorporated these indices into fully adjusted models to assess its independent association with mortality outcomes, we were unable to adequately account for the potential confounding effects of several pivotal inflammatory biomarkers, such as hsCRP, IL-6, and fibrinogen. Because these variables were either not collected or not consistently available across all NHANES survey cycles. Consequently, their omission represents an important limitation of the present study. Previous studies have demonstrated that elevated levels of inflammatory biomarkers such as hsCRP, IL-6, and fibrinogen can influence the cardiovascular system through multiple mechanisms, including promoting endothelial dysfunction, activating coagulation pathways, and exacerbating chronic inflammation, thereby directly or indirectly increasing the risk of CVD and mortality outcomes (50, 51). Therefore, the absence of adjustment for these inflammatory markers may have introduced residual confounding, which may have led to an overestimation of the association between MAR and outcomes. Therefore, future studies should integrate datasets that include a broader range of key inflammatory biomarkers, enabling more effective control of potential confounding factors. This would allow for a more accurate assessment of the independent predictive value of MAR and further clarify its incremental role in clinical risk stratification.

Moreover, this study is based on NHANES data, which confers strong representativeness for the U.S. population. However, its generalizability to populations outside the United States—particularly those in low- and middle-income countries—may be limited. First, nutritional status and socioeconomic conditions differ substantially across low- and middle-income countries. Previous studies have demonstrated that in low-income regions, chronic protein–energy malnutrition and unbalanced dietary patterns can result in persistently lower serum albumin levels (52), in addition, the higher burden of chronic infections frequently observed in these settings may further alter the distribution of MAR, potentially resulting in systematic differences compared with U.S. populations. Second, cardiovascular disease risk factors vary substantially across countries. For example, the Global Burden of Disease 2021 study reported that although the incidence of CVD has been declining in high-income countries, cardiovascular risk continues to rise in many low- and middle-income countries, where risk factors are more strongly influenced by undernutrition, infectious diseases, and limited healthcare resources (53). In such settings, the dual burden of inflammation and malnutrition may further amplify the association between MAR and CVD. Therefore, future studies should consider conducting external validation in populations from low- and middle-income countries to evaluate the distributional characteristics and optimal thresholds of MAR, as well as to examine its predictive performance under diverse nutritional and inflammatory conditions.

Beyond the two major limitations discussed above, it should also be acknowledged that the diagnosis of CVD in this study relied on participants’ self-reports of physician assessments, which may not fully capture the true incidence of the disease. In addition, the retrospective design of this study may introduce bias and limits the ability to establish causal inference. Therefore, future research should adopt prospective study designs, incorporate more rigorous and standardized diagnostic criteria for CVD, and account for a broader range of potential confounding variables to enhance the generalizability and validity of the findings. Despite these limitations, the present study provides several notable strengths and important clinical implications. First, we demonstrated that higher levels of MAR are significantly associated with increased risks of CVD, all-cause mortality, and cardiovascular mortality, offering valuable insights for disease surveillance in clinical settings. Second, we evaluated the predictive potential of MAR for CVD and adverse outcomes, suggesting that MAR may serve as a promising biomarker for early risk identification. Third, the robustness and consistency of our findings were reinforced by multiple sensitivity analyses, including subgroup analyses, lag-time analyses, and quartile-based stratifications. Finally, MAR, as a novel composite index, is simple to calculate and readily applicable in routine practice, thereby providing a practical tool to guide cardiovascular risk management and holding substantial promise for widespread clinical implementation.

## 5 Conclusion

In conclusion, MAR may serve as an effective supplementary biomarker for assessing the presence and prognosis of CVD. It also holds potential for contributing to risk stratification for all-cause and cardiovascular mortality in the general population. Therefore, supervising and sustaining optimal MAR values in clinical practice could help improve CVD outcomes and potentially decrease the likelihood of both all-cause and cardiovascular mortality.

## Data Availability

Publicly available datasets were analyzed in this study. This data can be found here: NHANES (https://www.cdc.gov/nchs/nhanes/).
