# Supplementary material for: Association of the monocyte-to-albumin ratio with cardiovascular disease and with all-cause and cardiovascular mortality in the general population
Source: Front Cardiovasc Med. 2025 Sep 22;12:1645793. doi: 10.3389/fcvm.2025.1645793 (PMC12497811; doi:10.3389/fcvm.2025.1645793)
Supplement: Supplementary file 1 [file Datasheet1.pdf]

Supplementary Material

Abbreviations:

NDI	National Death Index
NHANES	National Health and Nutrition Examination Survey
CVD	Cardiovascular disease
MAR	Monocyte-to-albumin ratio
ALI	Advanced lung cancer inflammation index
NPAR	Neutrophil to serum albumin ratio
CLR	C-reactive protein to lymphocyte ratio
NLR	Neutrophil to lymphocyte ratio
SII	Systemic immune inflammation index
HALP	Hemoglobin, albumin, lymphocyte and platelet scores
MLR	Mononuclear lymphocyte ratio
BMI	Body mass index
PIR	Poverty income ratio
RCS	Restricted cubic splines
ROC	Receiver operating characteristic curve
CHF	Congestive heart-failure
CHD	Coronary heart disease
TC	Total cholesterol
COPD	Chronic obstructive pulmonary disease
HR	Hazard ratio
AUC	Area under the curve

tables S1 Calculation methods of various composite inflammatory indicators

$\text{MAR} = \frac{\text{Monocyte(1000 cells/μL)}}{\text{Albumin(g/dL)}}$
$\text{SII} = \frac{\text{Neutrophil (1000 cells/μL)×Platelets (1000 cells/μL)}}{\text{Lymphocyte(1000 cells/μL)}}$
$\text{ALI} = \text{BMI} \times \text{Albumin (g/dL)} \times \frac{\text{Lymphocyte (1000 cells/μL)}}{\text{Neutrophil(1000 cells/μL)}}$
$\text{NLR} = \frac{\text{Neutrophil (1000 cells/μL)}}{\text{Lymphocyte(1000 cells/μL)}}$
$\text{HALP score} = \text{hemoglobin (g/dL)} \times \text{albumin (g/dL)} \times \frac{\text{Lymphocyte (1000 cells/μL)}}{\text{Platelets (1000 cells/μL)}}$
$\text{NAR} = \frac{\text{Neutrophil (1000 cells/μL)}}{\text{Albumin(g/dL)}}$

Table S2 The analysis of the association between MAR and CVD based on MAR quartile grouping

Variables	Model 1[3]		Model 2[4]		Model 3[5]	
	[1]OR (95%CI[2])	*P*-value	OR (95%CI)	*P*-value	OR (95%CI)	*P*-value
**MAR**	1.36 (1.26 ~ 1.47)	< 0.001	1.25 (1.16 ~ 1.34)	< 0.001	1.09 (1.03 ~ 1.16)	0.004
Quartile 1	1.00 (Reference)		1.00 (Reference)		1.00 (Reference)	
Quartile 2	1.16 (0.95 ~ 1.41)	0.157	1.10 (0.90 ~ 1.35)	0.354	0.99 (0.79 ~ 1.25)	0.961
Quartile 3	1.68 (1.39 ~ 2.04)	< 0.001	1.49 (1.23 ~ 1.81)	< 0.001	1.25 (1.01 ~ 1.54)	0.048
Quartile 4	2.54 (2.09 ~ 3.08)	< 0.001	2.03 (1.67 ~ 2.48)	< 0.001	1.39 (1.12 ~ 1.73)	0.003
P for trend	< 0.001		< 0.001		< 0.001	

In the correlation analysis, MAR was both standardized and categorized into quartiles for further analysis.

[1]OR, odds ratio; [2]95% Cl, 95% confidence interval.

[3]Model 1: No covariates were adjusted.

[4]Model 2: Adjusted for age, sex, and race.

[5]Model 3: Adjusted for age, sex, race, marital status, education level, BMI, PIR, TC, alcohol use, smoking status, hypertension, DM, COPD, and cancer.

Table S3 Cox regression of the link between MAR and all-cause mortality and CVD-cause mortality in the general population based on MAR quartile grouping

Mortality type/ Variable	Model 1[3] [1]HR (95%CI[2])	*P*-value	Model 2[4] HR (95%CI)	*P*-value	Model 3[5] HR (95%CI)	*P*-value
**All-cause mortality**						
Quartile 1	1.00 (Reference)		1.00 (Reference)		1.00 (Reference)	
Quartile 2	1.05 (0.87 - 1.26)	0.630	1.00 (0.84 - 1.20)	0.990	0.93 (0.78 - 1.12)	0.463
Quartile 3	1.35 (1.11 - 1.63)	0.002	1.19 (0.98 - 1.44)	0.086	1.07 (0.88 - 1.31)	0.484
Quartile 4	2.12 (1.82 - 2.46)	< 0.001	1.66 (1.42 - 1.94)	< 0.001	1.28 (1.09 - 1.52)	0.003
P for trend		< 0.001		< 0.001		< 0.001
**Cardiovascular mortality**						
Quartile 1	1.00 (Reference)		1.00 (Reference)		1.00 (Reference)	
Quartile 2	1.03 (0.76 - 1.39)	0.847	0.96 (0.72 - 1.30)	0.811	0.91 (0.67 - 1.23)	0.536
Quartile 3	1.52 (1.09 - 2.12)	0.013	1.27 (0.90 - 1.80)	0.174	1.19 (0.84 - 1.68)	0.324
Quartile 4	2.46 (1.86 - 3.27)	< 0.001	1.77 (1.32 - 2.38)	< 0.001	1.42 (1.05 - 1.91)	0.022
P for trend		< 0.001		< 0.001		0.002

In the correlation analysis, MAR underwent transformation into categorical classifications through quartile stratification.

[1]HR, hazard ratio; [2]95% Cl, 95% confidence interval.

[3]Model 1: No covariates were adjusted.

[4]Model 2: Adjusted for age, sex, and race.

[5]Model 3: Adjusted for age, sex, race, marital status, education level, BMI, PIR, TC, alcohol use, smoking status, hypertension, DM, COPD, and cancer.

Table S4 Cox regression of the association between monocyte percentage to albumin ratio and all-cause mortality and CVD-cause mortality including participants who had CVD history at baseline.

**Mortality type/ Variable**	**Model1**		**Model2**		**Model3**	
	**HR (95%CI)**	***P*-value**	**HR (95%CI)**	***P*-value**	**HR (95%CI)**	***P*-value**
**All-cause mortality**						
Tertile 1	1.00 (Reference)		1.00 (Reference)		1.00 (Reference)	
Tertile 2	1.12 (0.96 - 1.32)	0.150	1.02 (0.86 - 1.20)	0.846	0.94 (0.79 - 1.11)	0.444
Tertile 3	1.94 (1.70 - 2.23)	<.001	1.53 (1.33 - 1.76)	<.001	1.22 (1.06 - 1.41)	0.005
P for trend		<.001		<.001		<.001
**Cardiovascular mortality**						
Tertile 1	1.00 (Reference)		1.00 (Reference)		1.00 (Reference)	
Tertile 2	1.29 (0.97 - 1.72)	0.084	1.12 (0.83 - 1.49)	0.459	1.03 (0.77 - 1.39)	0.831
Tertile 3	2.40 (1.84 - 3.12)	<.001	1.74 (1.31 - 2.30)	<.001	1.41 (1.06 - 1.87)	0.018
P for trend		<.001		<.001		0.004

HR: Hazard Ratio, CI: Confidence Interval

Model1: No covariates were adjusted.

Model2: Adjust: age, sex, ethnicity.

Model3: Adjust: Adjusted for age, sex, ethnicity, education level, family poverty income ratio, marital status, smoking status, alcohol use, self-reported cancer, self-reported COPD, hypertension, total cholesterol level, duration of diabetes, body mass index, CVD.

Table S5 Cox regression of the association between monocyte percentage to albumin ratio and all-cause mortality and CVD-cause mortality with further adjustment of several biomarkers.

**Mortality type/ Variable**	**Model1**		**Model2**		**Model3**	
	**HR (95%CI)**	***P*-value**	**HR (95%CI)**	***P*-value**	**HR (95%CI)**	***P*-value**
All-cause mortality						
Tertile 1	1.00 (Reference)		1.00 (Reference)		1.00 (Reference)	
Tertile 2	1.12 (0.96 - 1.32)	0.150	1.02 (0.86 - 1.20)	0.846	0.95 (0.80 - 1.13)	0.585
Tertile 3	1.94 (1.70 - 2.23)	<.001	1.53 (1.33 - 1.76)	<.001	1.22 (1.05 - 1.42)	0.008
P for trend		<.001		<.001		<.001
Cardiovascular mortality						
Tertile 1	1.00 (Reference)		1.00 (Reference)		1.00 (Reference)	
Tertile 2	1.29 (0.97 - 1.72)	0.084	1.12 (0.83 - 1.49)	0.459	1.06 (0.78 - 1.44)	0.698
Tertile 3	2.40 (1.84 - 3.12)	<.001	1.74 (1.31 - 2.30)	<.001	1.39 (1.05 - 1.83)	0.023
P for trend		<.001		<.001		0.006

HR: Hazard Ratio, CI: Confidence Interval

Model1: No covariates were adjusted.

Model2: Adjust: age, sex, ethnicity.

Model3: Adjust: Adjusted for age, sex, ethnicity, education level, family poverty income ratio, marital status, smoking status, alcohol use, self-reported cancer, self-reported COPD, hypertension, total cholesterol level, duration of diabetes, body mass index, CVD, NLR, NAR, ALI, SII, HALP.

Table S6 Cox regression of the association between monocyte percentage to albumin ratio and all-cause mortality and CVD-cause mortality excluding individuals with less than 2 years of follow-up

Mortality type/ Variable	Model 1[3]		Model 2[4]		Model 3[5]	
	[1]HR (95%CI[2])	*P*-value	HR (95%CI)	*P*-value	HR (95%CI)	*P*-value
**All-cause mortality**						
Tertile 1	1.00 (Reference)		1.00 (Reference)		1.00 (Reference)	
Tertile 2	1.19 (1.01 - 1.42)	0.047	1.08 (0.90 - 1.29)	0.403	1.01 (0.84 - 1.22)	0.895
Tertile 3	1.88 (1.62 - 2.17)	< 0.001	1.49 (1.28 - 1.73)	< 0.001	1.21 (1.04 - 1.41)	0.014
P for trend		< 0.001		< 0.001		0.003
**Cardiovascular mortality**						
Tertile 1	1.00 (Reference)		1.00 (Reference)		1.00 (Reference)	
Tertile 2	1.31 (0.97 - 1.76)	0.078	1.14 (0.84 - 1.54)	0.412	1.08 (0.79 - 1.47)	0.619
Tertile 3	2.24 (1.69 - 2.97)	< 0.001	1.63 (1.22 - 2.20)	0.001	1.37 (1.02 - 1.83)	0.034
P for trend		< 0.001		< 0.001		0.015

[1]HR, hazard ratio; [2]95% Cl, 95% confidence interval.

[3]Model 1: No covariates were adjusted.

[4]Model 2: Adjusted for age, sex, and race.

[5]Model 3: Adjusted for age, sex, race, marital status, education level, BMI, PIR, TC, alcohol use, smoking status, hypertension, DM, COPD, and cancer.
